# In Memoriam of Marcello Bronstein

**DOI:** 10.20945/2359-3997000000619

**Published:** 2023-03-13

**Authors:** Julio Abucham

**Affiliations:** 1 Universidade Federal de São Paulo Escola Paulista de Medicina Unidade Neuroendócrina Brasil Unidade Neuroendócrina, Escola Paulista de Medicina, Universidade Federal de São Paulo

Finer still to join in laughing Underneath a silken heaven Lying back amid the grasses

Join with friends in cheerful laughing, Showing our white teeth together.

Am I right? let's lie in quiet;

Am I wrong? let's join in laughing And in being aggravating, Aggravating, loudly laughing, Till we reach the grave together.

“Among Friends. An Epilog” From: Human, all too human Friedrich Nietzsche (1)

We all lost a great friend. That loss was huge and premature in view of Marcello´s, enormous disposition to life in all senses. All those who knew him know how intense he was in his commitment to family, patient care, research, and teaching. All those qualities, but not only those qualities, made him the unique and virtuous human being he was. His talent and achievements have been well and deservedly praised by his many colleagues and friends here and abroad and will continue to be remembered for a long time.

On a more personal note, my contribution to his memory will focus in Marcello as my friend. Marcello and I became increasingly close friends since we first met, a long time ago, and as time went by we became brothers. When Silvia became his wife, she also became my sister from the very first time we met. And then came Ana, their beloved daughter, who became a dear niece.

Our friendship was built through our affinities in several matters including Medicine, Philosophy, Literature, Music, and so on. We constantly exchanged ideas and I learned a lot from him and I like to think that he also learned something from me. In his last year with us, I had the privilege to talk to him almost daily until that became no longer possible. Then Marcello left us, and that was a very hard reality that we all had to face. For our long and heartfelt friendship, I soon realized that Marcello is the one who stayed with me, not the one that was gone.

As I think of Marcello and our friendship, I realize that beyond all those things that we loved to share and which contributed to build our relationship, there is still something I cannot easily grasp. That missing element, so difficult to define, could perhaps be understood by answering a single but enigmatic question: how two souls recognize each other and become friends and brothers for life?

In reality, friendship has already been extensively studied and discussed by many philosophers over the centuries. Aristotle claimed that in true friendship, friends love each other for their own sake, and they wish good things for each other. This kind of friendship, he continues, is only possible between “good people similar in virtue” because only those are capable of loving another person for that person´sown sake(1). Nietzsche, in his unorthodox conception of friendship, thought that healthy friends work to refine him or her own self – and one another indirectly – in such a way they become similar to the “übermensch”, who Nietzsche valued as the being with most character and strength(2).

However close to what friendship is, should be, or means, those definitions fail to reach the quintessence of friendship and thus do not solve the enigma of the missing element. To my perception, no one has expressed it so concisely and beautifully as Montaigne did when asked to explain his exemplary friendship with Étienne De La Boétie:

-“Parce que c´etait lui, parce que c´etait moi”.

The pain and sorrow of Marcello´s departure will last for a long time because we all loved him. Nonetheless, no matter how big that loss is, let us not allow that the sadness of Marcello´s departure be greater than the happiness of having known him and having been his friend.

Thank you Marcello.
